# Case 5/2017 - A 28-Year-Old Woman with Cor Pulmonale Due to Pulmonary Hypertension Secondary to Chronic Pulmonary Thromboembolism

**DOI:** 10.5935/abc.20170148

**Published:** 2017-10

**Authors:** Jussara de Almeida Bruno, Rafael Amorim Belo Nunes, Paulo Sampaio Gutierrez, Vera Demarchi Aiello

**Affiliations:** Instituto do Coração (InCor) HC-FMUSP, São Paulo, SP - Brazil

**Keywords:** Pulmonary Heart Disease, Pulmonary, Hypertension, Pulmonary Embolism/complications, Risk Factors, Respiratory Insufficiency

The patient is a 28-year-old female, who presented with dyspnea on minimum exertion and dry cough.

The patient reported being asymptomatic until one year ago, when she had an episode of retrosternal pain followed by syncope, requiring admission to the intensive care unit, being then diagnosed with pulmonary thromboembolism (PTE).

Her technetium-99m diethylenetriaminepentaacetic acid (^99m^Tc-DTPA) radioaerosol inhalation lung scintigraphy (May 21, 2008) revealed marked hypoventilation of the left lung and retention of the radiotracer in the right peri-hilar region, suggestive of a parenchymal process. The use of ^99m^TC human albumin macroaggregates (^99m^TC MAA) revealed no perfusion in the left lung and perfusion defects in the right lung base.

Computed tomography (acute phase) with contrast suggested thrombosis of the left pulmonary artery.

The patient was referred for treatment at InCor.

On her first visit (Jul 8, 2008), she complained of dyspnea on milder than usual exertion and dry cough. She denied smoking, and reported being on oral contraception until the time of the PTE. Her obstetrical history revealed one gestation with normal delivery and no abortion.

Her physical examination showed heart rate (HR) of 80 bpm and blood pressure (BP) of 120/80 mm Hg. Her pulmonary auscultation showed reduced breath sound intensity in the left lung. Her cardiac auscultation was normal, as was her abdominal examination. There was edema (+/4+) in the left lower limb. Her pulses were palpable and symmetrical. Her peripheral capillary oxygen saturation (SpO_2_) was 90%. She was on warfarin, and her INR was 2.4.

Her laboratory tests (Jul 17, 2008) were as follows: glycemia, 70 mg/dL; creatinine, 0.81 mg/dL; potassium, 5.4 mEq/L; sodium, 141 mEq/L; hemoglobin, 17 g/dL; hematocrit, 53%; MCV, 91 fL; leukocytes, 12900/mm^3^ (65% neutrophils, 1% eosinophils, 29% lymphocytes and 5% monocytes); platelets, 341000/mm^3^; PT (INR), 2.4; APTT (rel), 1.17; normal urinalysis; homocysteine, 7.5 µmol/L. The lupus anticoagulant test was negative, and mutant prothrombin, absent. The anticardiolipin antibody test was negative, as were the antinuclear factor (ANF HEp-2; Anti-SM) and ANCA antibody tests.

Her echocardiogram (Sept 16, 2008) revealed the following diameters: aorta, 29 mm; left atrium, 30 mm; right ventricle, 34 mm; left ventricle (D/S), 39/23 mm; septal and posterior wall thickness, 8 mm. Left ventricular ejection fraction (LVEF) was 73%, left ventricular relaxation was abnormal, and ventricular septal motion, atypical. The right ventricle was markedly hypokinetic, and the valves, normal. The systolic pulmonary artery pressure was estimated as 50 mm Hg.

Computed tomography angiography of the pulmonary arteries (24 Sept 2008) revealed chronic PTE with occlusion of the left branch of the pulmonary artery.

Selective pulmonary angiography (Dec 17, 2008) showed occlusion at the origin of the left pulmonary artery. The right pulmonary artery was dilated and patent, and there was contrast stop at the level of the anterior basal branches of the lower lobe and branches of the middle lobe.

Spirometry revealed forced expiratory volume in 1 second (FEV_1_) of 71% of the predicted value, and forced vital capacity (FVC) of 68% of the predicted value, being the ventilatory disorder classified as mild.

Furosemide (40 mg) was prescribed, and warfarin, maintained. Surgical treatment of chronic thromboembolism by use of pulmonary endarterectomy was considered.

The dyspnea progressed to minimum exertion, being then accompanied by precordial pain and weight loss of 6 kg over 1 year. The patient was then hospitalized.

On physical examination (Mar 24, 2009), she was tachypneic (respiratory rate of 28 bpm), cyanotic and hydrated. Her HR was 100 bpm, and blood pressure, 110/80 mm Hg. Her weight was 69.7 kg, and height, 1.59 m. Her pulmonary auscultation revealed reduced breath sound intensity in the lung bases, worse at the right side. On cardiac auscultation, there was increased intensity of the pulmonary component of the second cardiac sound, and neither accessory sounds nor murmurs were heard. The abdomen was difficult to exam due to the patient’s dyspnea. Her left lower limb showed hard edema. Her pulses were normal and symmetrical. Her SpO_2_ was 84%, even with the use of an O_2_ catheter (5 L/min).

Her laboratory tests (Mar 25, 2009) were as follows: hemoglobin, 16.5 g/dL; hematocrit, 50%; MCV, 100 fL; leukocytes, 5000/mm^3^ (5% band neutrophils, 47% segmented neutrophils, 1% eosinophils, 42% lymphocytes and 5% monocytes); platelets, 229000/mm^3^; ESR, 1 mm; glucose, 68 mg/dL; urea, 26.1 mg/dL; creatinine, 0.94 mg/dL; sodium, 142 mEq/L; potassium, 4.7 mEq/L; AST, 21 U/L; ALT, 40 U/L; calcium, 4.4 mEq/L; phosphorus, 4.5 mg/dL; magnesium, 1.5 mEq/L; DHL, 238 u/L; CRP, 2.4 mg/L; BNP, 463 pg/mL; INR, 2.6; APTT (rel), 1.22.

Her ECG (Mar 29, 2009) revealed sinus rhythm, HR of 100 bpm, PR = 160 ms, dQRS = 80 ms, right atrial overload (P = 4 mV; SÂP = +60º) and right ventricular overload (SÂQRS = +120º forward, qR in V1).

Her echocardiogram (Mar 26 and 30, 2009) showed the following diameters: aorta, 29 mm; left atrium, 32; right ventricle, 40/45 mm; left ventricle, 40/26 mm. The ventricular septum and posterior wall thickness was 9 mm, and the LVEF, 65%. Left ventricular systole was normal, and the filling pattern showed relaxation impairment. The right ventricle was hypertrophic and severely hypokinetic. The valves had no changes. Systolic pulmonary artery pressure was estimated as 64 mm Hg.

The dyspnea and hypoxemia worsened, and the patient required orotracheal intubation. Nitric oxide, milrinone and cefepime were initiated (Mar 30, 2009).

Her laboratory tests (Mar 30, 2009) were as follows: urea, 39 mg/dL; creatinine, 0.92 mg/dL; glucose, 87 mg/dL; potassium, 4.2 mEq/L; sodium, 140 mEq/L; BNP, 510 pg/mL; INR, 1.8; TTPA (rel), 1.27; arterial lactate, 267 mg/dL. Arterial blood gas analysis revealed: pH, 7.41; pCO_2_, 23.5 mm Hg; pO_2_, 48.7 mm Hg; SatO_2_, 82%; HCO_3_, 17.2 mEq/L; and base excess (-) 3.2 mEq/L

Two hours after intubation, the patient had a cardiac arrest with pulseless electrical activity, which was initially reversed, but recurred few minutes later, and the patient died (Mar 31, 2009, 2h45min).

## Clinical aspects

We report the case of a 28-year female patient denying any previous morbidity, who had acute PTE and progressively developed significant functional impairment and signs suggestive of chronic PTE during follow-up until death.

Venous thromboembolism (VTE) is the third most frequent cause of cardiovascular disease in the general population, with an annual incidence of 100 to 200 cases per 100000 inhabitants, acute PTE being its most severe clinical presentation.^1^ The prevalence and incidence of spontaneous VTE in young adults are low, but increase significantly in the presence of risk factors, such as oral contraception use, obesity and thrombophilia, especially in associations. The use of oral contraceptives, such as estrogens/progestogens, increases by 2 to 4 times the risk of venous thromboembolic events.^2^ Activated protein C resistance is attributed to a mechanism related to higher risk for VTE in patients on oral contraceptives. In our case, the patient had been on regular use of oral contraceptives until the first event, but there is no information on their formulation. Obesity is considered a risk factor, increasing by 2.4 times the risk for VTE in obese individuals as compared to non-obese individuals.^3^ When associating obesity and oral contraceptive use simultaneously, the risk for VTE increases by 10 times.^4^ Significant thrombophilias, such as deficiencies in protein C, protein S and antithrombin, homozygosity for factor V Leiden and prothrombin gene mutation increase in up to 7 times the risk for venous thromboembolic events in patients on oral contraceptives.^5^ During the patient’s follow-up, certain thrombophilias, such as prothrombin gene mutation, hyperhomocysteinemia and antiphospholipid syndrome, were excluded, but neither factor V Leiden nor deficiency in natural anticoagulants were investigated.

The incidence of chronic PTE is heterogeneous, ranging from 0.4% to 9.1% of the patients after an acute embolic event in different studies.^6^ Its etiology is little known, being related to genetic and ethnic factors.^7^ Hypercoagulable states, such as clotting factor VIII elevation and presence of antiphospholipid antibodies, are related to thromboembolic pulmonary hypertension.^8^ Mortality related to recurrent PTE 3 to 6 months after anticoagulant therapy is approximately 0.4% per year, partially depending on the presence or absence of comorbidities. Patients with acute PTE, who develop systolic pulmonary hypertension (levels > 50 mm Hg), that is not solved in the first weeks, have worse prognosis. In addition, the incidence of death due to recurrent PTE or chronic pulmonary hypertension within the first 3 years after anticoagulant treatment discontinuation ranges from 1% to 3%.^9^

Our patient maintained significant pulmonary hypertension and right ventricular dysfunction according to the findings from both the echocardiography in September 2009, and the computed tomography angiography of the pulmonary arteries and the pulmonary angiography suggesting chronic occlusion of the left pulmonary artery despite the anticoagulant therapy instituted. During outpatient clinic follow-up, between September and December 2008, the possibility of surgical treatment was considered. Assessment for pulmonary thromboendarterectomy in patients with chronic PTE should be early, even in patients with non-limiting symptoms, because surgery can prevent irreversible vasculopathy. The decision to perform the procedure should consider whether the pulmonary artery anatomy is favorable, presence of hemodynamic and ventilatory abnormalities, comorbidities associated, and the patient’s will. In specialized centers, the mortality related to pulmonary thromboendarterectomy in low-risk patients is around 1.3%.^10^ In patients not eligible for surgical treatment and those maintaining pulmonary hypertension after the procedure, pharmacological treatment with the following pulmonary vasodilators should be considered: riociguat (soluble guanylate cyclase stimulator) and intravenous prostanoids, such as eprostinil and treprostinil, in critical patients. Phosphodiesterase inhibitors, such as sildenafil and tadalafil, and endothelin receptor antagonists, such as bosentan, can be alternatives to treatment.^11^

**Figure 1:**
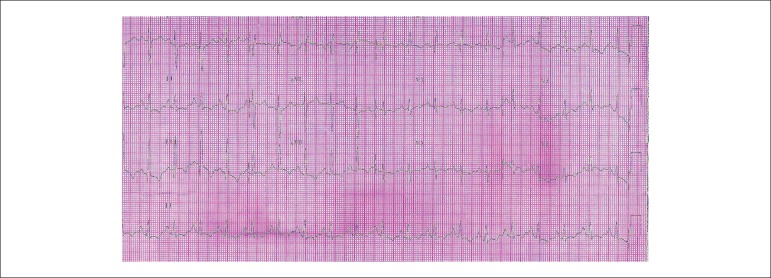
ECG: Sinus rhythm, right atrial overload, SÂQRS +120º, right ventricular overload.

The patient developed progressive dyspnea with important functional impairment until hospitalization in March 2009. She had the following factors of poor prognosis: advanced functional class (III/IV, according to the WHO classification); right ventricular systolic dysfunction; signs of overload of the right chambers (Figure 1); and lack of specific treatment (pharmacological or surgical). Her echocardiogram revealed increased right ventricular dimensions and elevated systolic pulmonary artery pressure as compared to previous measurements, in addition to persistence of important right ventricular dysfunction. It is worth noting the significant respiratory failure and hypoxemia even when using oxygen supplementation via catheter, which required orotracheal intubation for mechanical ventilation. Despite those measures, the patient had a cardiac arrest with pulseless electrical activity, probably related to refractory respiratory failure. Regarding the causes of decompensation and death, we considered the course of the underlying disease, with progressive aggravation of pulmonary arterial hypertension and right ventricular dysfunction, in addition to the likelihood of a new acute pulmonary thromboembolic event. **(Jussara de Almeida Bruno, MD, and Rafael Amorim Belo Nunes, MD)**

**Diagnostic hypothesis:** respiratory failure and hemodynamic collapse due to chronic thromboembolic pulmonary arterial hypertension and right ventricular dysfunction, and possible recurrence of acute pulmonary thromboembolism. **(Jussara de Almeida Bruno, MD, and Rafael Amorim Belo Nunes, MD)**

## Postmortem examination

**Figure 2:**
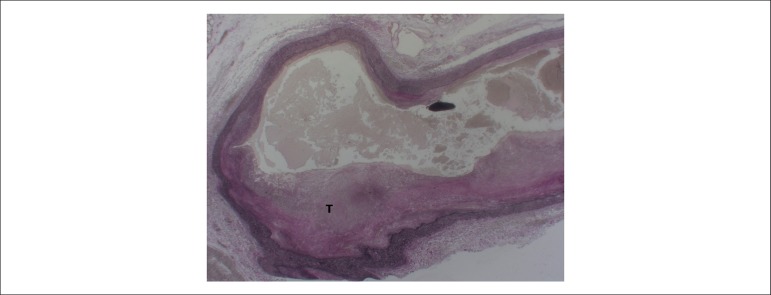
Microscopic section of o a central pulmonary artery showing partial occlusion by an organizing thrombus (T). Verhoeff stain; Objective magnification = 1X.

**Figure 3:**
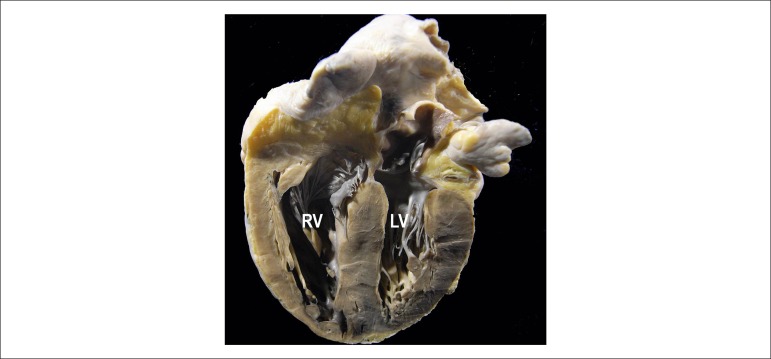
Gross aspect of the heart, frontal section, showing cor pulmonale, characterized by hypertrophy and dilatation of the right ventricle (RV), whose dimensions are close to those of the left ventricle (LV).

**Figure 4:**
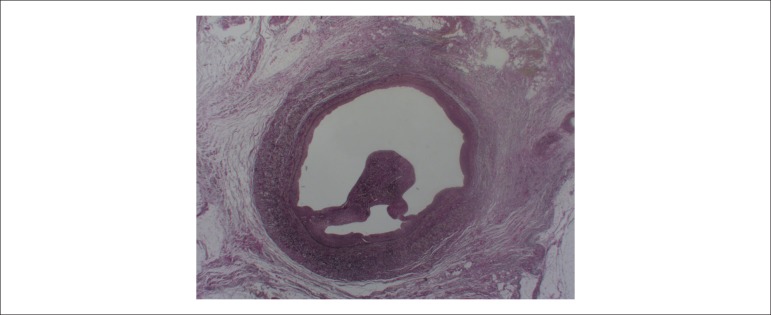
Microscopic section of the left iliac vein showing phlebosclerosis and organized thrombosis. Verhoeff stain; Objective magnification = 1X.

**Figure 5:**
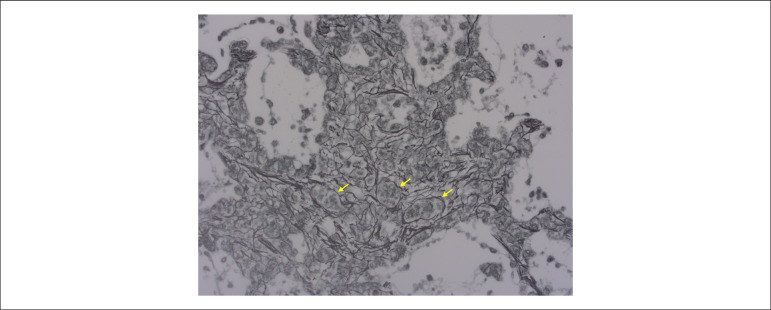
Microscopic section of the lung showing an area with capillary hemangiomatosis, characterized by the presence of more than one layer of capillaries (some indicated by the arrows) in alveolar septa. Reticulin stain; Objective magnification = 1X.

**Figure 6:**
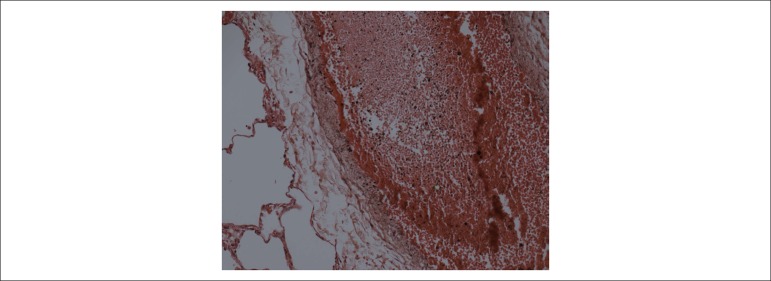
Microscopic section of an intrapulmonary arterial branch showing severe congestion, not conclusive of recent thromboembolism. Hematoxylin-Eosin; Objective magnification = 20X.

Not even the postmortem examination could clarify the major issues of this patient’s disease. The major findings were: partial occlusion of the left pulmonary artery (Figure 2); *cor pulmonale* (Figure 3); phlebosclerosis of the left iliac vein (Figure 4); focal areas similar to pulmonary capillary hemangiomatosis (Figure 5); and severe pulmonary congestion, with blood in larger vessels and questionable recent thromboembolism (Figure 6). The causes of neither chronic thromboembolism nor phlebosclerosis could be determined, and it was not certain whether the pulmonary vessels really had thromboemboli that would explain the sudden worsening of the patient’s condition and her death. The bone marrow pattern was normal to age. **(Prof. Paulo Sampaio Gutierrez, MD)**

**Anatomopathological diagnoses:** Major disease: chronic pulmonary thromboembolism.

**Cause of death:** undetermined (questionable recent thromboembolism). (**Prof. Paulo Sampaio Gutierrez, MD**)

## Comments

Neither the underlying disease nor the cause of death were determined, but the anatomopathological findings confirmed the clinical, echocardiographic and imaging diagnoses: the patient had chronic pulmonary thromboembolism, and signs of organized peripheral venous thrombosis.

Therefore, her thrombophilic condition, whose nature was not clarified even with the postmortem examination, was evident. Some thrombophilic conditions are as follows: collagen diseases, such as lupus and antiphospholipid antibody syndrome; hematological disorders; and postsplenectomy state. Apparently, lupus was ruled out based on the laboratory tests, but there was no time for a comprehensive clinical investigation.

In chronic pulmonary thromboembolism, the histopathological findings usually differ between central and peripheral arteries. Thrombi in central elastic arteries usually organize as intimal thickenings of varied degrees, which extend to the hilar branches.^12^ Surgical endarterectomy is aimed at resecting those thickenings, re-establishing local circulation. In smaller arteries, thromboses can organize as a re-channeling with multiple vascular lumens, named “colander lesion”, which should not be mistaken for the classic plexiform lesion.

However, in peripheral pulmonary arteries, the changes are usually similar to those found in the idiopathic form of pulmonary arterial hypertension and in the Eisenmenger syndrome, reflecting vascular remodeling in response to increased flow and shear stress in the distal portions of the vascular bed of the central arterial branches that were not obstructed by thrombosis.^13^ Those changes include mainly hypertrophy of the arterial tunica media and concentric proliferation of the intima.

In our case, it is worth noting the relatively mild remodeling of the peripheral pulmonary arteries, with mild hypertrophy of the tunica media and few foci of intimal thickening. In addition, the pattern known as pulmonary capillary hemangiomatosis was observed, a finding not usually described in the thromboembolic condition. That occurred in foci, being characterized by the presence of capillary proliferation in alveolar septa, in more than one layer, as opposed to the normal aspect of one single layer. That type of lesion has been mainly described in association with pulmonary veno-occlusive disease (absent in our case),^14^ but also in some other forms of pulmonary vascular disease^15^ or as an incidental necropsy finding.^16^ Its meaning is uncertain, but seems more often related to pulmonary venous hypertensive conditions. (**Prof. Vera Demarchi Aiello, MD**)

**Section editor:** Alfredo José Mansur (ajmansur@incor.usp.br)

**Associated editors:** Desidério Favarato (dclfavarato@incor.usp.br)

Vera Demarchi Aiello (anpvera@incor.usp.br)
